# Infection—Ready Mode of Preventing

**Published:** 1871-12

**Authors:** J. Sartin


Infection—Ready Mode of Preventing.—There is perhaps no plan of preventing infection so ready as the production of sulphurous acid by the combustion of sulphur. To disinfect a bed, whilst the patient is temporarily removed from it, pass a copper warming-pan into the bed, containing a few live embers and a little sulphur. The pan should be moved about during the ignition of the sulphur. By burning sulphur in an open vessel, closets, carriages, passages, and vacated chambers of the sick, may be easily disinfected. Clothing may be lightly sponged over or sprinkled with water containing a little well mingled sulphur, and then ironed with a flat-iron heated to a temperament which will cause volatilization of the sulphur without burning the linen.—Mr. J. Sartin.—*Braithwaite.*

